# Asthma Associated Cytokines Regulate the Expression of SARS-CoV-2 Receptor ACE2 in the Lung Tissue of Asthmatic Patients

**DOI:** 10.3389/fimmu.2021.796094

**Published:** 2022-01-17

**Authors:** Fatemeh Saheb Sharif-Askari, Swati Goel, Narjes Saheb Sharif-Askari, Shirin Hafezi, Saba Al Heialy, Mahmood Yaseen Hachim, Ibrahim Yaseen Hachim, Bassam Mahboub, Laila Salameh, Mawada Abdelrazig, Eman Ibrahim Elzain, Saleh Al-Muhsen, Mohamed S. Al-Hajjaj, Elaref Ratemi, Qutayba Hamid, Rabih Halwani

**Affiliations:** ^1^ Sharjah Institute of Medical Research, College of Medicine, University of Sharjah, Sharjah, United Arab Emirates; ^2^ College of Medicine, Mohammed Bin Rashid University of Medicine and Health Sciences, Dubai, United Arab Emirates; ^3^ Meakins-Christie Laboratories, McGill University, Montreal, QC, Canada; ^4^ Department of Clinical Sciences, College of Medicine, University of Sharjah, Sharjah, United Arab Emirates; ^5^ Rashid Hospital, Dubai Health Authority, Dubai, United Arab Emirates; ^6^ Immunology Research Lab, Department of Pediatrics, College of Medicine, King Saud University, Riyadh, Saudi Arabia; ^7^ Department of Pediatrics, College of Medicine, King Saud University, Riyadh, Saudi Arabia; ^8^ Jubail-Industrial College, Department of Chemical and Process Engineering Technology, Jubail-Industrial City, Saudi Arabia; ^9^ Prince Abdullah Ben Khaled Celiac Disease Chair, department of pediatrics, Faculty of Medicine, King Saud University, Riyadh, Saudi Arabia

**Keywords:** COVID-19, SARS-CoV-2, ACE2, TMPRSS2, asthma, cytokines, IL-19, IL-17

## Abstract

It is still controversial whether chronic lung inflammation increases the risk for COVID-19. One of the risk factors for acquiring COVID-19 is the level of expression of SARS-CoV-2 entry receptors, ACE2 and TMPRSS2, in lung tissue. It is, however, not clear how lung tissue inflammation affects expression levels of these receptors. We hence aimed to determine the level of SARS-CoV-2 receptors in lung tissue of asthmatic relative to age, gender, and asthma severity, and to investigate the factors regulating that. Therefore, gene expression data sets of well-known asthmatic cohorts (SARP and U-BIOPRED) were used to evaluate the association of ACE2 and TMPRSS2 with age, gender of the asthmatic patients, and also the type of the underlying lung tissue inflammatory cytokines. Notably, ACE2 and to less extent TMPRSS2 expression were upregulated in the lung tissue of asthmatics compared to healthy controls. Although a differential expression of ACE2, but not TMPRSS2 was observed relative to age within the moderate and severe asthma groups, our data suggest that age may not be a key regulatory factor of its expression. The type of tissue inflammation, however, associated significantly with ACE2 and TMPRSS2 expression levels following adjusting with age, gender and oral corticosteroids use of the patient. Type I cytokine (IFN-γ), IL-8, and IL-19 were associated with increased expression, while Type II cytokines (IL-4 and IL-13) with lower expression of ACE2 in lung tissue (airway epithelium and/or lung biopsies) of moderate and severe asthmatic patients. Of note, IL-19 was associated with ACE2 expression while IL-17 was associated with TMPRSS2 expression in sputum of asthmatic subjects. *In vitro* treatment of bronchial fibroblasts with IL-17 and IL-19 cytokines confirmed the regulatory effect of these cytokines on SARS-CoV-2 entry receptors. Our results suggest that the type of inflammation may regulate ACE2 and TMPRSS2 expression in the lung tissue of asthmatics and may hence affect susceptibility to SARS-CoV-2 infection.

## Introduction

More than two years into this global pandemic crisis, there are still controversies surrounding the association of chronic respiratory diseases such as asthma and risk of infection with SARS-CoV-2 virus, the cause of coronavirus disease (COVID-19). As such, early report suggested that patients with asthma are at increased risk for hospitalization and other severe outcomes from COVID-19 (1, 2), while recent reports have shown that the risk of intubation in patients with asthma is similar to those without asthma (3). Therefore, the matter of which factors of asthma may increase the risk for COVID-19 infection is still a topic of debate and require further investigation.

The fact that SARS-CoV-2 enters lung cells *via* ACE receptor (4) stimulated huge efforts to identify the factors that may directly or indirectly regulate level of expression of this receptor in the airway cells, and hence the risk for COVID-19 infectivity. In the context of asthma, the focus was mainly on the effect of selective asthma-specific cytokines such as IL-13 on the airway expression level of ACE2 (5, 6). IL-13 was found to be associated with reduced expression level of ACE2 in airway epithelial cells of allergic asthma (5). In line with this, our group found that Type II cytokines (IL-13, IL-5, and IL-4) were associated with reduced expression levels of ACE2 and TMPRSS2 in nasal tissues of eosinophilic chronic rhinosinusitis patients (7). Therefore, better characterization of the effect of asthma-specific cytokines on ACE2 expression and hence on the infectivity and severity of COVID-19 infection is needed.

Asthma is now widely accepted as a complex disease involving distinct but interrelating inflammatory pathways. The type of lung tissue inflammation differ based on asthma phenotype, endotype (Type II high or low) and severity (8). Knowing the suggested ability of Type I and II cytokines to regulate ACE2 and TMPRSS2 expression (6, 9), it was intriguing to understand how asthma-related cytokines and the type of lung tissue inflammation may regulate SARS-CoV-2 receptors in the different phenotypes of asthma. Moreover, knowing that age and gender may determine risk for increased COVID-19 severity, we have also determined whether ACE2 and TMPRSS2 lung tissue expression levels differs relative to age and gender of asthmatic patients.

## Methods

### Gene Expression Datasets

Publicly available gene expression datasets of adult asthmatics lung tissue available at the National Center for Biotechnology Information Gene Expression Omnibus (NCIB GEO, http://www.ncbi.nlm.nih.gov/geo), and the European Bioinformatics Institute (EMBL-EBI, https://www.ebi.ac.uk) were used. Quality control (QC) was performed before data analysis (10, 11). The datasets included are of bronchial brushing (GSE43696) (12, 13), bronchial biopsies (GSE76227) (14), and sputum (GSE147880) of patients with moderate to severe asthma. Dataset of IL-13 treated (GSE37693) (15) as well as IFN-γ, IL-17, and IL-4 treated (GSE148829) (9) airway epithelial cells were also used.

GSE43696 dataset of the Severe Asthma Research Program (SARP) cohort (12, 13): bronchial brushings were obtained *via* bronchoscope-directed epithelial brushings from patients with moderate (n = 50) and severe asthma (n = 38), and also from healthy adult controls (n = 20). Fresh isolated bronchial brushings comprised of more than 90% epithelial cells were analyzed by gene expression array profiling. Demographic data of cohort are added in **Supplementary Table 1**.

GSE76227 dataset of the Unbiased BIOmarkers in Prediction of REspiratory Disease outcomes (U-BIOPRED) Project (14): bronchial biopsy tissues from patients with moderate (n = 33) and severe asthma (n = 58) were collected. Column-based extraction of total RNA was performed followed by gene expression array profiling. Characteristics of patients are added in **Supplementary Table 2**.

GSE147880 dataset of the U-BIOPRED and Australian Newcastle severe asthma cohorts: induced sputum was collected from mild/moderate (n = 18) and severe (n = 16) asthmatic subjects. RNA was then extracted, and gene expression array profiling was performed. Detailed demographic data of cohort are added in **Supplementary Table 3**.

GSE37693 dataset (15): RNA was isolated from primary culture airway epithelial cells grown at an air–liquid interface, and was treated with or without IL-13 for 21 days.

GSE148829 dataset (9): RNA was isolated from the commonly-used human bronchial cell line BEAS-2B (ATCC, CRL-9609) treated or not with IFN-g, IL-17, or IL-4.

### Cell Culture

Primary bronchial fibroblasts were isolated from endobronchial tissue biopsies obtained from 3 healthy controls and severe asthmatic donors, as described previously (16, 17). The cells isolated from each donor were studied separately. The clinical characteristics of the donors are deposited in **Supplementary Table 4**. The cells were cultured at confluence in 6 well flat-bottom in complete media (Dulbecco’s modified Eagle’s media (Sigma) supplemented with 10% fetal bovine serum (FBS), 2 mM L-glutamine, 100 units/ml of penicillin, and 100 ng/ml streptomycin). The cells were then treated, or not, with 25 ng/ml of recombinant IL-17 (Sigma), 25, 50, and 100 ng/ml of recombinant human IL-19 (9286-IL-19, R&D), or 100 ng/ml denatured IL-19 for 8 h. Denatured IL-19 was heat denatured at 100°C for 10 min. Total RNA was extracted using Trizol (Invitrogen) according to manufacturer instructions. For cDNA amplification 5× Hot FirePol EvaGreen qRT-PCR SuperMix (Solis Biodyne) was used and RT-qPCR was performed in QuantStudio 3 Real-Time PCR System (Applied Biosystems).

### Western Blot Assay

The protein concentrations were measured using the BCA protein assay reagent kit (ThermoScientific Pierce BCA Protein Assay Kit). The cells were lysed using 10× RIPA Buffer (Abcam) after supplementation with 1× Protease Inhibitor Cocktail (Sigma-Aldrich) and 1 mM phenylmethylsulfonyl fluoride (Sigma-Aldrich). Twenty micrograms total proteins were separated using 8% gels. The proteins were transferred onto a nitrocellulose membrane (Bio-Rad), blocked in skimmed milk for 1 h at room temperature, incubated overnight at 4°C with antibodies specific to ACE2 (Cell Signaling Technology). β-Actin (Cell Signaling Technology) was used as loading controls. The blots were developed using the Clarity Western ECL Substrate (Bio-Rad) in the ChemiDoc Touch Gel Imaging System (Bio-Rad). Image Lab software (Bio-Rad) was used to detect and quantify the protein bands.

### Analysis Procedures

Microarray data (CEL files) were pre-processed with Robust Multi-Array Average (RMA) using R software (18). The raw microarray data was normalized, and log transformed. The RNA-seq data was normalized using voom method (19). The fold change of differential expressed genes were carried out using *Limma* Bioconductor package (20, 21). Association with ACE2 expression and asthma related-cytokine (22) were evaluated using linear regression models adjusted for age, gender, smoking, atopy status, and use of oral corticosteroids. The model was adjusted for age and gender in airway epithelium; for age, gender and oral corticosteroids use in lung tissue datasets; and for age, gender, smoking, atopic status, and oral corticosteroid use in sputum dataset. All selected variables were tested for multicollinearity to avoid any strong correlation between the variables. The presence of collinearity was examined by the evaluation of variance inflation factors and magnitude of standard errors.

For treatment of fibroblasts with IL-17 or IL-19, all results were expressed as fold change relative to untreated bronchial fibroblasts. The following primers were used: human ACE2, forward, 5’-3’: CCACGAAGCCGAAGACCTG, and reverse, 5’-3’: GGCAGACCATTTGTCCCCAGC; human TMPRSS2, forward, 5’-3’: CCGCTGGCCTACTCTGGAAGTT,and reverse, 5’-3’: GTGTGACACGCCATCACACCAGT; human 18s, forward, 5’-3’: TGACTCAACACGGGAAACC, and reverse, 5’-3’: TCGCTCCACCAACTAAGAAC. Gene expression was analyzed using the Comparative Ct (ΔΔCt) method after normalization to the housekeeping gene 18 s rRNA. Analyses were performed using R software (v 3.0.2), SPSS Version 26 (IBM Corporation, Chicago, USA), and Graphpad Prism 8 (GraphPad Software Inc., San Diego, USA). For all analyses, P-values <0.05 were considered significant.

## Results

### SARS‐CoV‐2 Receptor ACE2, and TMPRSS2 Expression is Upregulated in Airway Epithelium and Bronchial Biopsies of Asthmatic Patients

**Figure 1:**
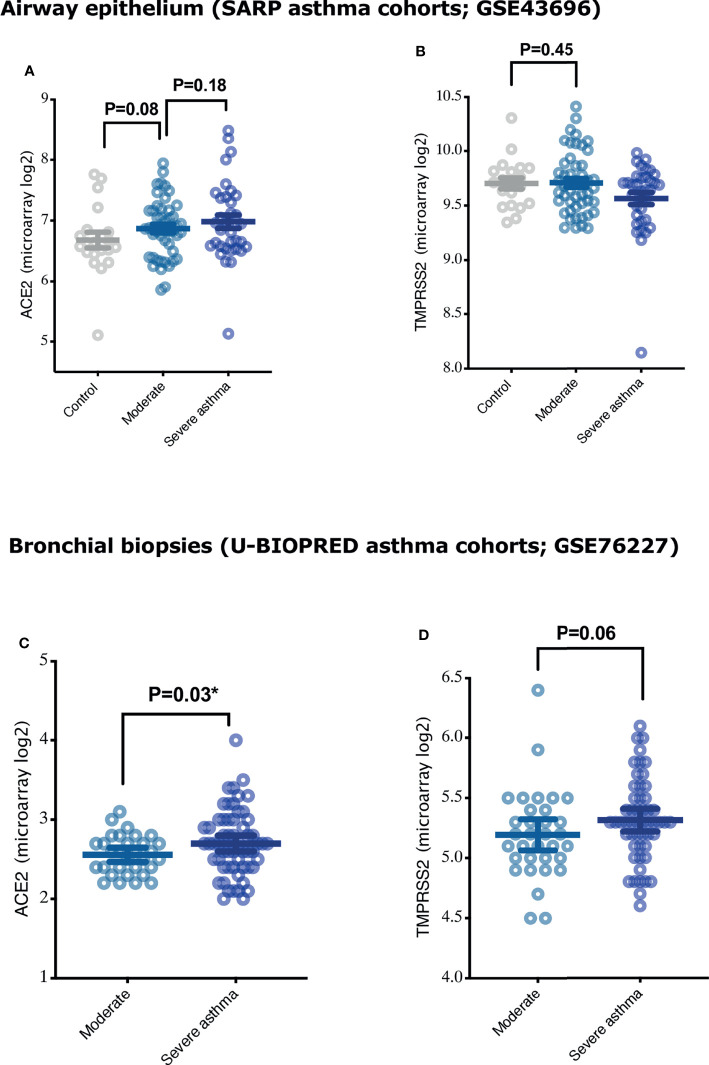
Expression of COVID-19 entry genes, ACE2 and TMPRSS2, in airway epithelium and bronchial biopsies of asthmatics with different severity categories. **(A, B)** The expression of ACE2 and TMPRSS2 in airway epithelium of healthy controls (n = 20), moderate (n = 50), and severe asthmatics (n = 38) **(C, D)** The expression of ACE2 and TMPRSS2 in the bronchial biopsies of moderate (n = 33) and severe asthmatics (n = 58). Two-way comparison was done using unpaired t-test or Mann–Whitney U test, depending on the skewness of the data. *P < 0.05.

It has been reported that the level of expression of SARS COV-2 receptors may determine the level of susceptibility of the lung tissue to SARS-CoV-2 infection (4). We hence determined whether ACE2 and TMPRSS2 are differentially expressed in the airway epithelium and lung tissue of large cohort of asthmatics (airway epithelium of SARP asthma cohort; GSE43696, and bronchial biopsies of U-BIOPRED cohort; GSE76227). We found that the expression of ACE2 was slightly upregulated in the airway epithelium of asthmatics compared to healthy controls (**Figure 1A**), with a trend of increase in ACE2 expression relative to asthma severity (**Figure 1A**). However, ACE2 was significantly increased in bronchial biopsies of severe compared to moderate asthmatics (**Figure 1C**). Additionally, no difference was observed in expression of TMPRSS2 in airway epithelium of asthmatics compared to healthy controls (**Figure 1B**), while there was a trend of increase in TMPRSS2 expression in bronchial biopsies of severe compared to moderate asthmatics (**Figure 1D**).

### Lung Tissue Expression of ACE2, but not TMPRSS2, was Affected by the Age of Asthmatic Patients

**Figure 2:**
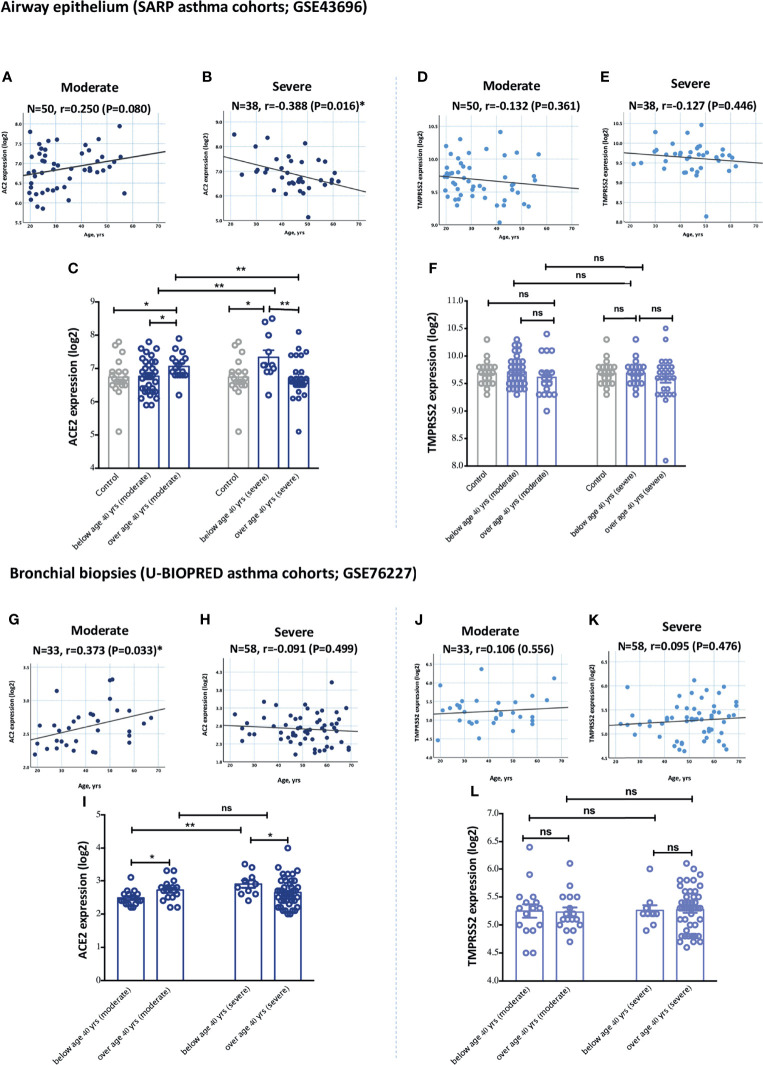
Gene expression of ACE2 and TMPRSS2 in airway epithelium and bronchial biopsies of moderate and severe asthmatics relative to age. **(A, C)** The expression level of ACE2 in airway epithelium of moderate asthmatics below and above 40 years of age **(B, C)** The expression of ACE2 in airway epithelium of severe asthmatics below and above 40 years of age. **(D, F)** The expression levels of TMPRSS2 in airway epithelium of moderate asthmatics below and above 40 years of age **(E, F)** The expression of TMPRSS2 in airway epithelium of severe asthmatics below and above 40 years of age. **(G, I)** The expression levels of TMPRSS2 in bronchial biopsies of moderate asthmatics below and above 40 years of age **(H, I)** The expression of ACE2 in bronchial biopsies of severe asthmatics below and above 40 years of age. **(J, L)** The expression levels of TMPRSS2 in bronchial biopsies of moderate asthmatics below and above 40 years of age **(K, L)** The expression of TMPRSS2 in bronchial biopsies of severe asthmatics below and above 40 years of age. Correlation between ACE2 or TMPRSS2 gene expression level and individual’s age was measured using Pearson’s correlation coefficient with a two‐sided test for significance (P < 0.05 significant). Two-way comparison was done using unpaired t-test or Mann–Whitney U test, depending on the skewness of the data. ns, non-significant. *P < 0.05, **P < 0.01.

Aging is believed to increase the risk, and also contribute to the pathogenesis, of both asthma and COVID-19 disease. Indeed, elderly patients are more prone to develop severe COVID-19 disease compared to younger population (1, 23). Aging is also believed to affect asthma pathogenesis (24). We hence investigated whether ACE2 is more expressed in elderly asthmatics compared to younger patients. The expression of ACE2 was significantly different relative to age in moderate and severe asthmatics. Age was characterized into above and below 40 years based on the average age of physician-diagnosed asthma in adults (25). Patients with moderate asthma who are above 40 years of age had significantly higher ACE2 expression levels in the airway epithelium (**Figures 2A, C**) and bronchial biopsies (**Figures 2G, I**) compared to younger patients. However, ACE2 levels in the lung tissue of severe asthmatics decreased with age. Severe asthmatic patients below 40 years of age had significantly higher expression of ACE2 in the airway epithelium (**Figures 2B, C**) and bronchial biopsies (**Figures 2H, I**) compared to those above 40 years old. This indicates that elderly with moderate asthma, and young patients with severe asthma have higher levels of ACE2 in their lungs compared to other asthmatic individuals. Similar trend of association between age and ACE2 expression in lung tissue was observed following further adjustment with gender and oral corticosteroids use (**Supplementary Tables 5**, **6**). On the contrary, the expression level of TMPRSS2 was approximately similar in airway epithelium and bronchial biopsies of both moderate and severe asthma subjects across different age groups (**Figures 2D–F**, for airway epithelium; and **Figures 2J–L**, for bronchial biopsies).

### Lung Expression of ACE2 and TMPRSS2 did not Differ Relative to Patient’s Gender

**Figure 3:**
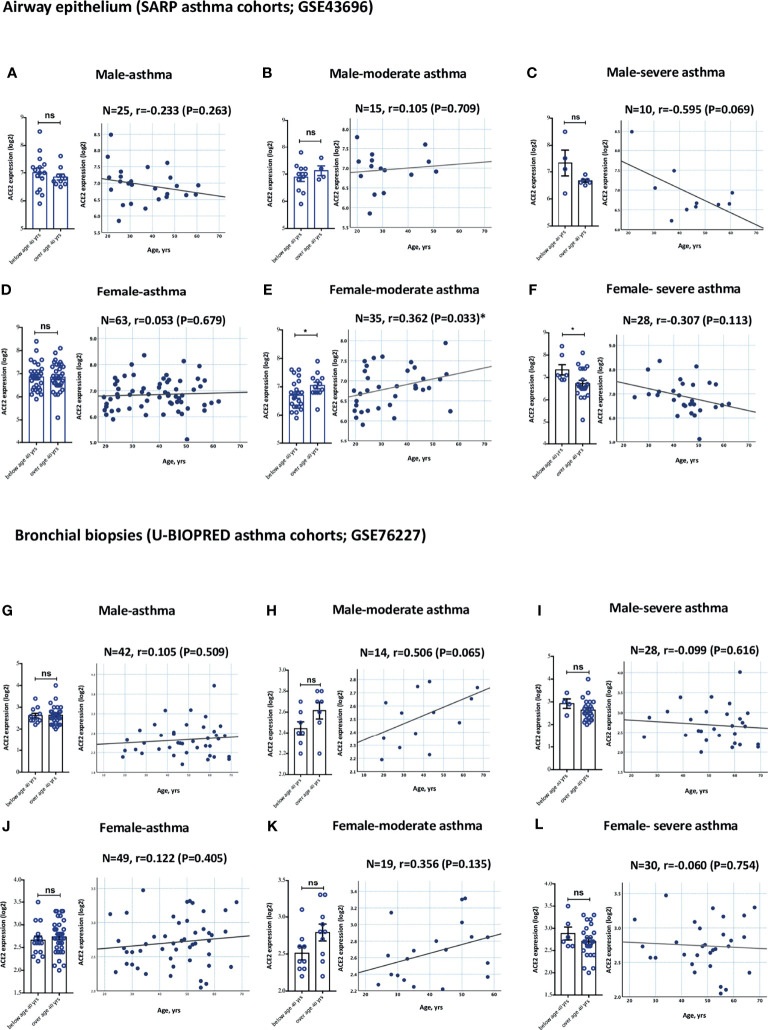
Gene expression of ACE2 in airway epithelium and bronchial biopsies of moderate and severe asthmatics relative to gender. **(A–C)** ACE2 expression level in AECs has an increasing trend in elderly males with moderate asthma and young males with severe asthma. **(D–F)** ACE2 expression level is significantly increased in AECs of elderly females with moderate asthma and young females with severe asthma. **(G–I)** ACE2 expression level in bronchial biopsies has an increasing trend in elderly males with moderate asthma and young males with severe asthma. **(J–L)** ACE2 expression level in bronchial biopsies has an increasing trend in elderly females with moderate asthma and young females with severe asthma. ns, non-significant. Correlation between ACE2 gene expression level and individual’s age was measured using Pearson’s correlation coefficient with a two‐sided test for significance (P <0.05 significant). Two-way comparison was done using unpaired t-test or Mann–Whitney U test, depending on the skewness of the data. ns, non-significant. *P < 0.05.

Gender is another variable that is believed to play a role in determining the clinical course of COVID-19 disease. We hence investigated whether gender has an effect on the expression of these receptors in the different asthma severity groups. No significant difference in the levels of ACE2 and TMPRSS2 expression relative to gender was observed in both groups of asthmatics (**Figures 3A, D** for airway epithelium of ACE2; **Figures 3G, J** for bronchial biopsies of ACE2; and **Supplementary Figure 1** for TMPRSS2). Both moderate asthmatic males and females aged 40 years and above had higher ACE2 expression level in their airway epithelium and bronchial biopsies compared to younger patients (**Figures 3B, E**; and **Figures 3H, K**, respectively); While both young severe asthmatic males and females below age of 40 years had higher ACE2 expression level in their airway epithelium and bronchial biopsies compared to older patients (**Figures 3C, F**; and **Figures 3I, L**, respectively).

### The Expression of ACE2 and TMPRSS2 Correlates With Asthma-Associated Inflammatory Cytokines

**Figure 4:**
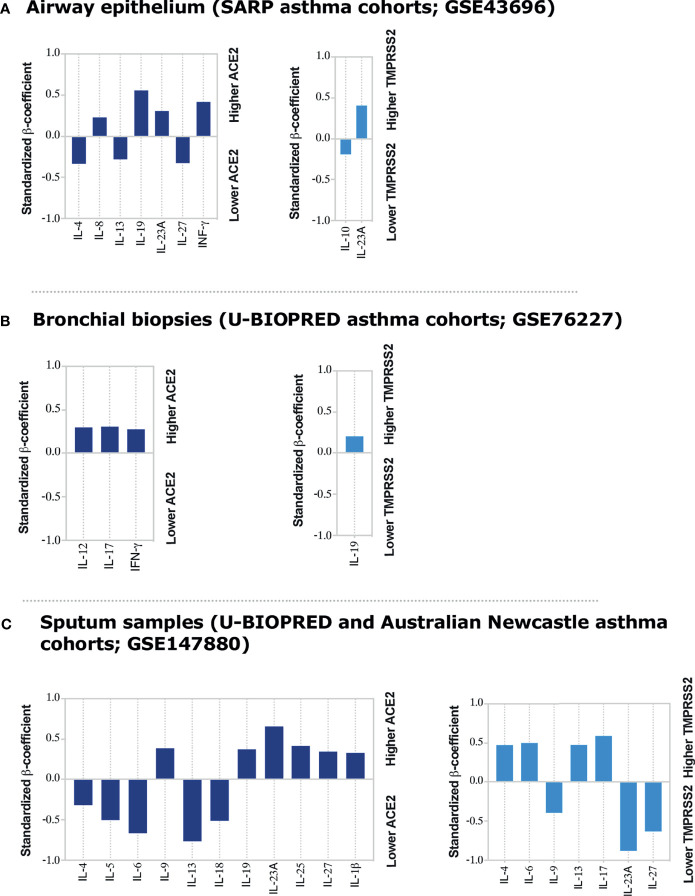
Inflammatory Cytokines associated with ACE2 and TMPRSS2 expression in airway epithelium, bronchial biopsies, and sputum of moderate and severe asthmatics. Linear regression model was used to determine correlations of ACE2 and TMPRSS2 with asthma-associated cytokines in **(A)** airway epithelium, **(B)** bronchial biopsies, and **(C)** sputum samples of moderate to severe asthma patients, respectively. The linear regression model was adjusted for age and gender in airway epithelium; for age, gender and oral corticosteroid use in lung tissue datasets; and for age, gender, smoking, and atopic status of asthmatic patients in sputum dataset. None of asthma patients in sputum dataset were on oral corticosteroid use. The standardized β-coefficient values were plotted to aid in interpretation of the degree of association of each cytokine with ACE2 or TMPRSS2 in respective tissues. All the bar charts showing significant association with P < 0.05.

IFN-γ (9) and Type II cytokines (6) have been suggested to regulate ACE2 expression. IFN-γ was shown to enhance the expression of ACE2 in nasal and bronchial epithelial cells (9). TGF-β also reduced the expression of ACE2 receptor in lung cells (26). Since most of these cytokines are associated with asthma pathogenesis, this suggest these inflammatory mediators could play a critical role in regulating ACE2 and TEMPRSS2 expression in the lung tissue of asthmatics. To investigate that, we determined the association between the type of inflammation, i.e., asthma-related inflammatory cytokines (22) and ACE2 and TEMPRSS2 expression levels. The gene expression levels of IL-2, IL-12, IL-27, IFN-γ, IL-4, IL-5, IL-13, IL-6, IL-8, IL-18, IL-1β, TNF-α, IL-17, IL-23A, IL-25, IL-9, IL-10, IL-19, and TGF-β in data set of airway epithelium, bronchial biopsies, and sputum of both moderate and severe asthmatic patients were tested for their association with ACE2 and TMPRSS2 gene expression. Notably, after adjusting for age, gender, smoking, atopy status, and use of oral corticosteroids we found a significant positive correlation between a range of these pro-inflammatory cytokines and ACE2 or TMPRSS2 expression levels in airway epitheliums, bronchial biopsies, and the sputum of moderate and severe asthma subjects (**Figure 4** and **Supplementary Tables 7–9**).

ACE2 correlated positively with IFN-γ, IL-8, IL-19, and IL-23A gene expression in airway epithelium (**Figure 4A**); with IL-12, IL-17, and IFN-γ gene expression, in bronchial biopsies (**Figure 4B**); and with IL-9, IL-19, IL-23A, IL-25, IL-27, and IL-1β gene expression in sputum (**Figure 4C**) of asthmatic subjects all to a significant level. On the contrary, the expression of ACE2 was inversely correlated with IL-4, IL-13, and IL-27 gene expression in the airway epithelium (**Figure 4A**); and with IL-4, IL-5, IL-6, IL-13, and IL-18 gene expression in sputum of asthmatic patients (**Figure 4C**). On the other hand, TMPRSS2 expression correlated positively with IL-23A and negatively with IL-10 gene expression in airway epithelium (**Figure 4A**); with IL-19 gene expression in bronchial biopsies (**Figure 4B**); and with IL-4, IL-6, IL-13, and IL-17 gene expression in sputum of asthmatic subjects (**Figure 4C**), all to a significant level. Its expression was, however, inversely correlated with IL-9, IL-23A, and IL-27 gene expression in the sputum of asthmatic subjects (**Figure 4C**). The association of ACE2 or TMPRSS2 with IL-2, TNF-α, and TGF-β gene expression was not significant in any of the tested tissues (**Supplementary Tables 7–9**).

**Figure 5:**
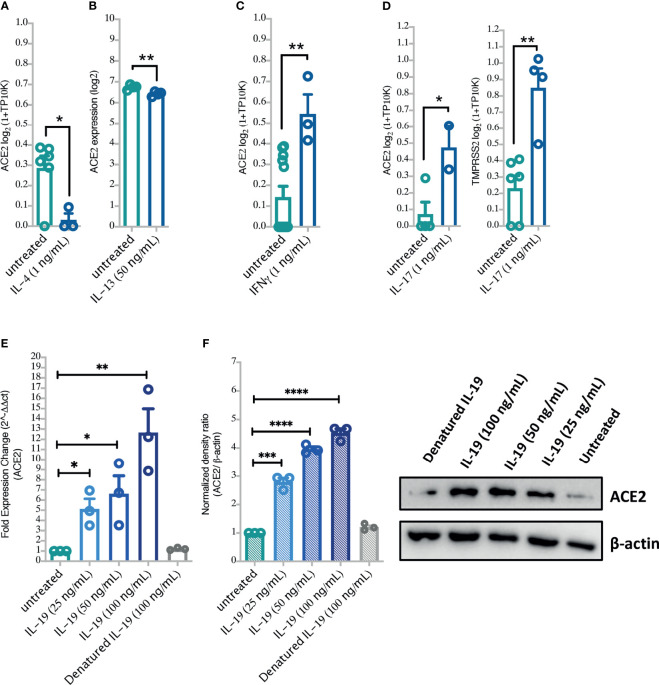
The effects of Type II cytokines, IFN-γ, and IL-19 stimulation on the lung cell expression levels of ACE2 and TMPRSS2. **(A–D)** The expression of ACE2 and TMPRSS2 following treatment of BEAS-2B cells with IL-4, IL-13, IFN-γ and IL-17 (GSE37693; for IL-13, and GSE148829 for IFN-**γ**, IL-17, and IL-4 treatment). **(E, F)** ACE2 mRNA and protein expression in primary human bronchial fibroblasts of severe asthmatic (n = 3) following *in vitro* stimulation with IL-19 cytokine. Blots were visualized on a BioRad ChemiDoc™ Touch Imager-ACE2; exposure time of 54 s (signal accumulation) and β-actin; exposure time of 0.4 s (Optimal Auto-exposure). Two-way comparison was done using unpaired t-test or Mann–Whitney U test, depending on the skewness of the data. *P < 0.05, **P < 0.01, ***P < 0.001, ****P < 0.0001.

Moreover, to confirm the suggested regulation of ACE2 by these cytokines, a data set of IL-4, IL-13, or IFN-γ stimulated airway epithelial cells was used. The differential expression of ACE2 was then assessed in these cells following cytokine stimulation. Treating healthy human airway epithelial cells with IL-4 or IL-13 significantly downregulated their expression of ACE2 (**Figures 5A, B**). Treatment with IFN-γ, on the other hand, upregulated ACE2 expression in these cells to a significant level (**Figure 5C**). Interestingly, stimulation of airway epithelial cells with IL-17 enhanced the expression of ACE2 and TMPRSS2 in these cells (**Figure 5D**).

Moreover, to confirm our *in silico* findings, we treated primary bronchial fibroblasts with different doses of IL-17 or/and IL-19 and assessed their ACE2 and TMPRSS2 expression. As expected, IL-17 treatment significantly increased expression of ACE2 and TMPRSS2 in a dose-dependent manner in both bronchial fibroblasts of severe asthma and healthy controls with no significant difference in their expression between the two groups (**Supplementary Figure 2**). We also treated bronchial fibroblasts of severe asthma with IL-19 and assessed their ACE2 and TMPRSS2 expression. Of interest, IL-19 treatment was associated with a significant increase in ACE2 and TMPRSS2 expression in a dose-dependent manner (**Figures 5E, F** and **Supplementary Figures 2**, **3**) in these cells. The combination treatment of IL-17 and IL-19 resulted in higher expression of ACE2 but not TMPRSS2 in these cells (**Supplementary Figure 2**).

Furthermore, we have assessed the relation between expression levels of IL-19 or IL-17 and ACE2 or TMPRSS2 in the nasopharyngeal swabs of large cohort of COVID-19 patients (n = 430; GSE152075) and notably, found that expression levels of these cytokines of nasopharyngeal swab in COVID-19 positively correlated with ACE2 or TMPRSS2 expression levels (**Supplementary Figures 3A, B**).

## Discussion

Although epidemiological studies did not identify asthma as one of the top risk factor for severe COVID19—severe illnesses (1, 23), the chronic lung inflammation and airway tissue remodeling characteristic of this disease may predispose to worse outcomes following COVID-19 infection. It is believed that uncontrolled SARS-CoV-2 viral replication is driving the development of severe symptoms. The level of SARS-CoV-2 receptor expression may hence be a critical determinant of increased lung tissue infectivity and viral tissue levels. In this report, upon screening the gene expression datasets of bronchial epithelium and bronchial biopsies of large cohorts of well-defined asthmatic patients, we observed that the expression of COVID-19 receptor, ACE2, is significantly upregulated in the lung tissue of young severe asthmatic patients, and in patients with moderate asthma but above 40 years of age. This was also apparent in the study by Lovinsky-Desir et al. (27) which studied the prevalence of asthma among hospitalized patients with COVID-19 and found asthma to be more common among younger patients hospitalized with COVID-19 compared to adults, suggesting asthma may be a risk for severe COVID-19 among the pediatric population.

Our further investigation revealed that the observed differential expression of ACE2 receptors within the different age and severity groups is associated with the type of the underlying lung tissue inflammation. Type I as well as IL-17 regulatory cytokines normally associated with neutrophilic asthma (IFN-γ, IL-8, IL-12, IL-17, IL-19, and IL23A) were correlated with an increase in ACE2 expression in airway epithelium and bronchial biopsies of asthmatic patients. Similar correlation was also observed with IL-9, IL-19, IL-23A, IL-25, IL-27, and IL-1β in sputum of these patients. On the contrary, Type II cytokines (IL-4, IL-5, and IL-13), normally associated with eosinophilic asthma, as well as IL-6, IL-18 and IL-27 were correlated with suppression of ACE2 expression. This finding is consistent with reports indicating that IL-13 cytokine is suppressive of ACE2 expression in both nasal and bronchial epithelial cells (5, 6). This further suggest that Type II low asthmatics may have higher expression of ACE2 in their lung airways compared to Type II high asthmatics. As such, young severe asthmatics with higher expression of ACE2 had lower expression of Type II cytokines (IL-4, IL-5, and IL-13) and higher expression of IL-19 and IL-17 in the airway epithelium and bronchial biopsies compared to their moderate counterparts (**Supplementary Figure 4**).

Moreover, it is noteworthy that IL-9 which is a Type II cytokine behaved differently from other Type II cytokines (IL-4, IL-5, and IL-13) and was associated with an increase in ACE2 expression in sputum of asthma patients. This may be due to the fact that IL-9 is a pleiotropic cytokine and produced by variety of cells like mast cells, Th2, Th17, and Th9 cells in different amount. The interaction of IL-9 with its receptor is critical for promoting the proliferation of inflammatory cells in the lungs through activating the JAK/STAT pathway and recruiting neutrophils and eosinophils (28). It was shown that IL-9 level was increased in SARS-CoV-2 infected human airway epithelial cultures compared to Mock treated cultures (29). Higher serum level of IL-9 was also reported in patients with COVID-19 pneumonia compared to those of healthy controls (30). This overall confirms the association of IL-9 with SARS-CoV-2 infection. However, further mechanistic studies are needed to confirm the association with SARS-CoV-2 cell receptor gene ACE2.

To our knowledge, this is the first report to highlight the correlation of IL-17 and IL-19 with SARS-CoV-2 receptors expression in the lung airways of asthmatic patients. The expression of IL-17 cytokine was associated with increased ACE2 and TMPRSS2 expression in the bronchial biopsies and sputum of asthmatic subjects, respectively. While IL-19 cytokine was mostly associated with increased ACE2 receptor in airway epithelium and sputum samples of asthma cohorts. We have also confirmed these correlations *in vitro*, by treating non-asthmatic and severe asthmatic bronchial fibroblasts with IL-17 and/or IL-19 recombinant cytokines. This resulted in an upregulation of ACE2 and/or TMPRSS2 expression levels in both non-asthmatic and severe asthmatic bronchial fibroblasts to a similar level. This comparable increase could be due to the fact that severe asthmatics were on oral corticosteroid treatment that has been shown to reduce ACE2 expression *in vitro* in bronchial fibroblasts (7). Reduced ACE2 and TMPRSS2 expression were also reported in sputum samples of asthmatics who were on prolonged used of inhaled corticosteroids (31). Therefore, to account for this effect of corticosteroids, the associations between the expression level of these receptors and different asthma-associated cytokines were adjusted for the use of corticosteroids in the airway epithelium, bronchial biopsies, and sputum samples of studied asthma cohorts (**Figure 4**).

Little is known about the biological function of IL-19. IL-19 was shown to be upregulated in the blood and saliva of asthmatics compared to control and to induce the expression of Th-2 cytokines in activated T cells (32, 33). Moreover, inhibition of IL-19 or blocking its receptor, IL-20R1, protected rodents from allergic lung inflammation (34). IL-19 was also reported to enhance the expression of IL-17 in airway epithelial cells (35), which may refer to its role in airway tissue modulating. We have previously shown that IL-19 receptors (IL-1R1 and IL-1R2) are upregulated in bronchial fibroblast following IL-19 treatment (33). Treating severe asthmatic bronchial fibroblasts with increasing concentration of IL-19 resulted in a relative increase in ACE2 expression suggesting a direct regulatory role of this cytokine on ACE2. Further investigations, however, is needed to determine the exact mechanism of how IL-19 regulate ACE2 expression.

Prior data indicated that ACE2 expression is upregulated in certain group of asthma patients such as men, African Americans, diabetes, and those who are on prolonged use of corticosteroids (31). Our findings suggest that this should not be generalized for all asthma patients. Asthma endotype and the type of underlying inflammation are factors that may regulate ACE2 expression in the lungs of these asthmatics, and hence need to be considered. More studies focused on chronic respiratory diseases, such as asthma, COPD, and other chronic respiratory diseases are needed to provide better understanding of the impact of chronic lung inflammation on COVID-19 susceptibility and disease severity. Our report suggests that Type I inflammation could enhances, while Type II may suppress ACE2 expression in the lung airway of asthmatics. Better characterization of the effect of these cytokines on ACE2 expression and hence on the infectivity and severity of COVID-19 infection is needed. Further interpretation of these relationships could identify novel therapeutic strategies to more effectively control the current COVID-19 pandemic.

Our study is limited in the fact that it is based on transcriptomic gene datasets which may not necessarily reflect the protein expression levels of cytokines and also the SARS-CoV-2 receptors. However, as is shown in **Figure 5** there was significant increase in the levels of ACE2 in bronchial fibroblasts upon treatment with IL-19. Another limitation is the fact that data from publicly available data sets do not include detailed patients’ clinical information.

## Conclusions

In summary, using datasets of a large cohort of asthmatic patients, with well characterized phenotypes and endotypes, we have shown that ACE2 is differentially expressed in the lungs of asthmatics, relative to the predominant type of inflammatory cytokines. The type of asthma endotype and the underlying inflammation need to be considered when evaluating ACE2 expression in asthmatics. Type II cytokines, IFN-γ, IL-17, and IL-19 could be crucial in regulating the expression of ACE2 and TMPRSS2 in the lung airway of asthmatics. This however, does not exclude the possibility that age related factors may also contribute to the observed difference in ACE2 expression between young and old patients. Further research is required to determine the impact of these factors on ACE2 lung expression and the implications on COVID19 severity.

## Data Availability Statement

Publicly available datasets were analyzed in this study. This data can be found here: All data presented are from publicly available datasets, as detailed in the manuscript. https://www.ncbi.nlm.nih.gov/geo/query/acc.cgi?acc=GSE43696 https://www.ncbi.nlm.nih.gov/geo/query/acc.cgi?acc=GSE76227 https://www.ncbi.nlm.nih.gov/geo/query/acc.cgi?acc=GSE147880 (Available upon request) https://www.ncbi.nlm.nih.gov/geo/query/acc.cgi?acc=GSE37693 https://www.ncbi.nlm.nih.gov/geo/query/acc.cgi?acc=GSE148829 https://www.ncbi.nlm.nih.gov/geo/query/acc.cgi?acc=GSE152075 RMA codes. https://github.com/Bioma-85/Asthma-biomarkers/blob/main/RMA.txt.

## Ethics Statement

Ethics approval for this study was obtained from by the Fonds de la Recherche en Santé du Québec Respiratory Health Network Tissue Bank (McGill University Health Centre/Meakins-Christie Laboratories Tissue Bank, Montreal, Canada). The patients/participants provided their written informed consent to participate in this study.

## Author Contributions

FS-A, NS-A, RH, and QH conceived and designed the experiments. FS-A, SG, and ShiH performed experiments. FS-A and NS-A analyzed the data. All authors contributed to the article and approved the submitted version.

## Funding

This research has been financially supported by the Tissue Injury and Repair (TIR) group operational grant (Grant code: 150317); the COVID-19 research grant (CoV19-0307); the Seed grant (Grant code: 2001090275); and by the collaborative research grant (Grant code: 2001090278) to RH, University of Sharjah, UAE; and by a Sandooq Al Watan Applied Research & Development grant to RH (SWARD-S20-007); by an Al Jalila Foundation Seed Grant (AJF202019); and by Prince Abdullah Ben Khalid Celiac Disease Research Chair, under the Vice Deanship of Research Chairs, King Saud University, Riyadh, Kingdom of Saudi Arabia.

## Conflict of Interest

The authors declare that the research was conducted in the absence of any commercial or financial relationships that could be construed as a potential conflict of interest.

## Publisher’s Note

All claims expressed in this article are solely those of the authors and do not necessarily represent those of their affiliated organizations, or those of the publisher, the editors and the reviewers. Any product that may be evaluated in this article, or claim that may be made by its manufacturer, is not guaranteed or endorsed by the publisher.

## Abbreviations

SARS-CoV-2, Severe Acute Respiratory Syndrome-Coronavirus-2; COVID-19, Coronavirus Disease 2019; ACE2, Angiotensin Converting Enzyme-2; TMPRSS2, Transmembrane Serine Protease 2; IL-23A, interleukin 23A; IL-8, interleukin 8; IL-6, interleukin 6; IL-17, interleukin 17; IL-13, interleukin 13; IL-4, interleukin 4; IL-19, interleukin 19; EGF, Epidermal growth factor; CCR5, C-C chemokine receptor type 5; CXCR3, C-X-C Motif Chemokine Receptor 3; TGF-β, Tumor growth factor-beta; IL-12, interleukin 12; IFN-γ, interleukin-gamma; TNF-α, Tumor necrosis factor-alpha; ICAM1, Intercellular Adhesion Molecule 1; CCR2, C-C chemokine receptor type 2; CCR3, C-C chemokine receptor type 3.
