# Supplementary material for: Asthma Associated Cytokines Regulate the Expression of SARS-CoV-2 Receptor ACE2 in the Lung Tissue of Asthmatic Patients
Source: Front Immunol. 2022 Jan 17;12:796094. doi: 10.3389/fimmu.2021.796094 (PMC8801531; doi:10.3389/fimmu.2021.796094)
Supplement: Supplementary file 1 [file DataSheet_1.pdf]

Asthma associated cytokines regulate the expression of SARS-CoV-2 receptor ACE2 in the lung tissue of asthmatic patients

Fatemeh Saheb Sharif-Askari[1], Swati Goel[1], Narjes Saheb Sharif-Askari[1], Shirin Hafezi[1], Saba Al Heialy[2,3], Mahmood Yaseen Hachim[2], Ibrahim Yaseen Hachim[1,4], Bassam Mahboub[1,5], Laila Salameh[1,5], Mawada Abdelrazig[5], Eman Ibrahim Elzain[5], Saleh Al-Muhsen[6,7], Mohamed S Al-Hajjaj[1,4], Elaref Ratemi[8], Qutayba Hamid [1,3,4], Rabih Halwani [1,4,9]

Supplementary Table 1. Demographics of subjects in study GSE43696

	**Healthy control (n=20)**	**Mild-moderate* asthma (n=50)**	**Severe asthma* (n=38)**	**P-value****
Age, mean (SD), years	33 (13)	33 (11)	44 (10)	0.001
Male gender (%)	9/20 (45)	15/50 (30)	10/38 (26.3)	NS

*There was no difference in the use of inhaled corticosteroids [1, 2].
**NS: non-significant, *P*-values of less than 0.05 were considered as statistically significant.

Supplementary Table 2. Demographics of subjects in study GSE76227

	**Mild-moderate asthma (n=33)**	**Severe asthma (n=58)**	**P-value***
Age, mean (SD), years	40 (13)	50 (12)	0.001
Male gender (%)	14/33 (42.4)	28/58 (48.3)	NS
Oral corticosteroids	0	24/58 (41%)	0.001

*NS: non-significant, *P*-values of less than 0.05 were considered as statistically significant.

Supplementary Table 3. Demographics of subjects in study GSE147880

	**Healthy control (n=10)**	**Mild-moderate asthma (n=18)**	**Severe asthma* (n=16)**	**P-value****
Age, mean (SD), years	43 (19)	53 (14)	58 (14)	NS
Male	2/10 (20%)	6/18 (33.3%)	9/16 (56.3%)	NS
Atopic status	6/10 (60%)	12/18 (66.7%)	12/16 (75%)	NS
Former smoker	1/10 (10%)	5/18 (27.8%)	11/16 (68.8%)	0.005

*None of the severe asthmatic were on oral corticosteroid use.
**NS: non-significant, *P*-values of less than 0.05 were considered as statistically significant.

Supplementary Table 4. Clinical characteristics of bronchial fibroblast donors

	**Non-asthma (n=3)**	**Severe asthma (n=3)**	**P-value***
Age, mean (SD), years	43 (12)	43 (8)	0.573
BMI, mean (SD)	31 (2)	30 (2)	0.971
Male	2/3	1/3	0.414
Smoker	0	0	-
Oral use of corticosteroids	0	2/3	-

* *P*-values of less than 0.05 were considered as statistically significant.

Supplementary Table 5. Association of age and ACE2 expression in the airway epithelium dataset (GSE43696)

**Variables**	**Association with *ACE2* (β coefficient)**	**P-value***
Age below 40 years-severe asthma	0.427	0.009
Age over 40 years-moderate asthma	0.308	0.031

Adjusted for age and gender.
* *P*-values of less than 0.05 were considered as statistically significant.

Supplementary Table 6. Association of age and ACE2 expression in the lung biopsies dataset (GSE76227)

**Variables**	**Association with *ACE2* (β coefficient)**	**P-value***
Age below 40 years-severe	0.250	0.07
Age above 40 years-moderate	0.379	0.028

Adjusted for age, gender and use of oral corticosteroids.
* *P*-values of less than 0.05 were considered as statistically significant.

Supplementary Table 7. Association of asthma-specific cytokines and airway epithelium *ACE2* and *TMPRSS2* expression in the GSE43696 cohort

Variables	Association with *ACE2* (β coefficient)	*P* value	Association with *TMPRSS2* (β coefficient)	*P* value*
IL2	0.092	0.396	-0.075	0.492
IL12	-0.043	0.701	-0.147	0.183
IL27	-0.330	0.002	-0.100	0.354
IFNg	0.418	0.000	-0.127	0.253
IL4	-0.337	0.002	-0.192	0.081
IL5	-0.093	0.399	0.008	0.944
IL13	-0.284	0.009	0.096	0.382
IL6	-0.144	0.186	-0.077	0.480
IL8	0.231	0.035	-0.074	0.501
IL18	-0.060	0.590	-0.243	0.025**
IL1B	0.050	0.652	-0.030	0.781
TNFα	0.162	0.147	-0.072	0.519
IL17	0.033	0.765	-0.011	0.922
IL23A	0.307	0.004	0.407	0.000
IL25	-0.125	0.262	0.009	0.933
IL9	-0.059	0.593	0.250	0.019**
IL10	0.034	0.730	-0.194	0.046*
IL19	0.557	0.000	0.204	0.057
TGFB	-0.211	0.053	-0.095	0.385

Adjusted for age and gender.
**P*-values of less than 0.05 were considered as statistically significant.
**Non-significant linear regression model.

-0.192 0.052

Supplementary Table 8. Association of asthma-specific cytokines and lung tissue *ACE2* and *TMPRSS2* expression in the GSE76227 cohort

Variables	Association with *ACE2* (β coefficient)	*P* value	Association with *TMPRSS2* (β coefficient)	*P*-value*
IL2	0.146	0.177	0.170	0.107
IL12	0.316	0.002	0.109	0.294
IL27	0.076	0.479	-0.121	0.251
IFNg	0.286	0.007	-0.078	0.460
IL4	0.060	0.581	-0.054	0.611
IL5	0.034	0.756	-0.184	0.082
IL13	0.194	0.066	0.119	0.250
IL6	-0.065	0.540	-0.085	0.412
IL8	0.073	0.497	0.039	0.711
IL18	0.038	0.725	-0.050	0.637
IL1B	0.143	0.185	0.202	0.055
TNFα	-0.036	0.734	0.021	0.842
IL17	0.324	0.002	0.034	0.745
IL23A	0.132	0.218	0.043	0.681
IL25	0.080	0.456	-0.096	0.358
IL9	-0.094	0.388	0.180	0.090
IL10	0.081	0.457	-0.039	0.719
IL19	0.037	0.727	0.208	0.043
TGFB	0.014	0.893	-0.001	0.990

Adjusted for age, gender, and oral corticosteroids use.
**P*-values of less than 0.05 were considered as statistically significant.

Supplementary Table 9. Association of asthma-specific cytokines and sputum *ACE2* and *TMPRSS2* expression in the GSE147880 cohort

Variables	Association with *ACE2* (β coefficient)	*P* value	Association with *TMPRSS2* (β coefficient)	*P*-value*
IL2	0.104	0.535	-0.157	0.393
IL12	-0.166	0.332	0.377	0.040*
IL27	0.339	0.038	-0.631	0.000
IFNg	0.041	0.811	-0.040	0.834
IL4	-0.318	0.048	0.476	0.006
IL5	-0.503	0.001	0.335	0.062
IL13	-0.766	0.000	0.471	0.012
IL6	-0.666	0.000	0.502	0.006
IL8	0.247	0.136	0.178	0.336
IL18	-0.512	0.002	0.078	0.688
IL1B	0.324	0.047	-0.054	0.773
TNFα	0.206	0.247	0.137	0.489
IL17	-0.259	0.100	0.586	0.000
IL23A	0.658	0.000	-0.878	0.000
IL25	0.418	0.006	0.075	0.674
IL9	0.382	0.019	-0.394	0.029*
IL10	0.065	0.672	0.061	0.715
IL19	0.368	0.027	-0.013	0.946
TGFB	-0.166	0.308	0.022	0.905

Adjusted for age, gender, smoking, atopic status, and oral corticosteroid use.
**P*-values of less than 0.05 were considered as statistically significant.
**Non-significant linear regression model.

References

1. Voraphani, N., et al., *An airway epithelial iNOS–DUOX2–thyroid peroxidase metabolome drives Th1/Th2 nitrative stress in human severe asthma.* Mucosal immunology, 2014. **7**(5): p. 1175-1185.
2. Li, X., et al., *Expression of asthma susceptibility genes in bronchial epithelial cells and bronchial alveolar lavage in the Severe Asthma Research Program (SARP) cohort.* Journal of Asthma, 2016. **53**(8): p. 775-782.
