# Supplementary material for: Asthma Associated Cytokines Regulate the Expression of SARS-CoV-2 Receptor ACE2 in the Lung Tissue of Asthmatic Patients
Source: Front Immunol. 2022 Jan 17;12:796094. doi: 10.3389/fimmu.2021.796094 (PMC8801531; doi:10.3389/fimmu.2021.796094)
Supplement: Supplementary file 2 [file DataSheet_2.pdf]

Asthma associated cytokines regulate the expression of SARS-CoV-2 receptor ACE2 in the lung tissue of asthmatic patients

Fatemeh Saheb Sharif-Askari[1], Swati Goel[1], Narjes Saheb Sharif-Askari[1], Shirin Hafezi[1], Saba Al Heialy[2,3], Mahmood Yaseen Hachim[2], Ibrahim Yaseen Hachim[1,4], Bassam Mahboub[1,5], Laila Salameh[1,5], Mawada Abdelrazig[5], Eman Ibrahim Elzain[5], Saleh Al-Muhsen[6,7], Mohamed S Al-Hajjaj[1,4], Elaref Ratemi[8], Qutayba Hamid [1,3,4], Rabih Halwani [1,4,9]

Airway epithelium (SARP asthma cohorts; GSE43696)

A

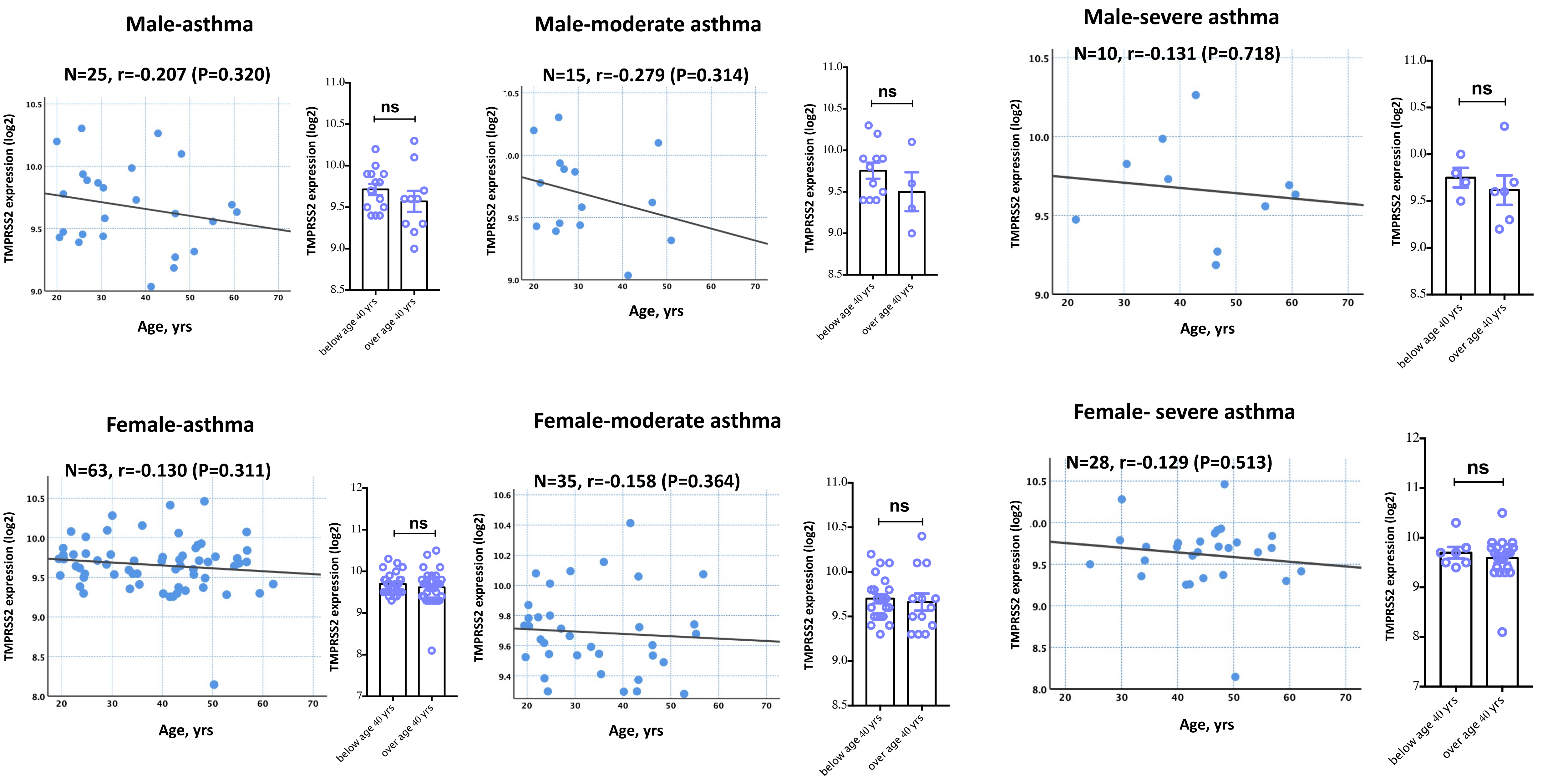

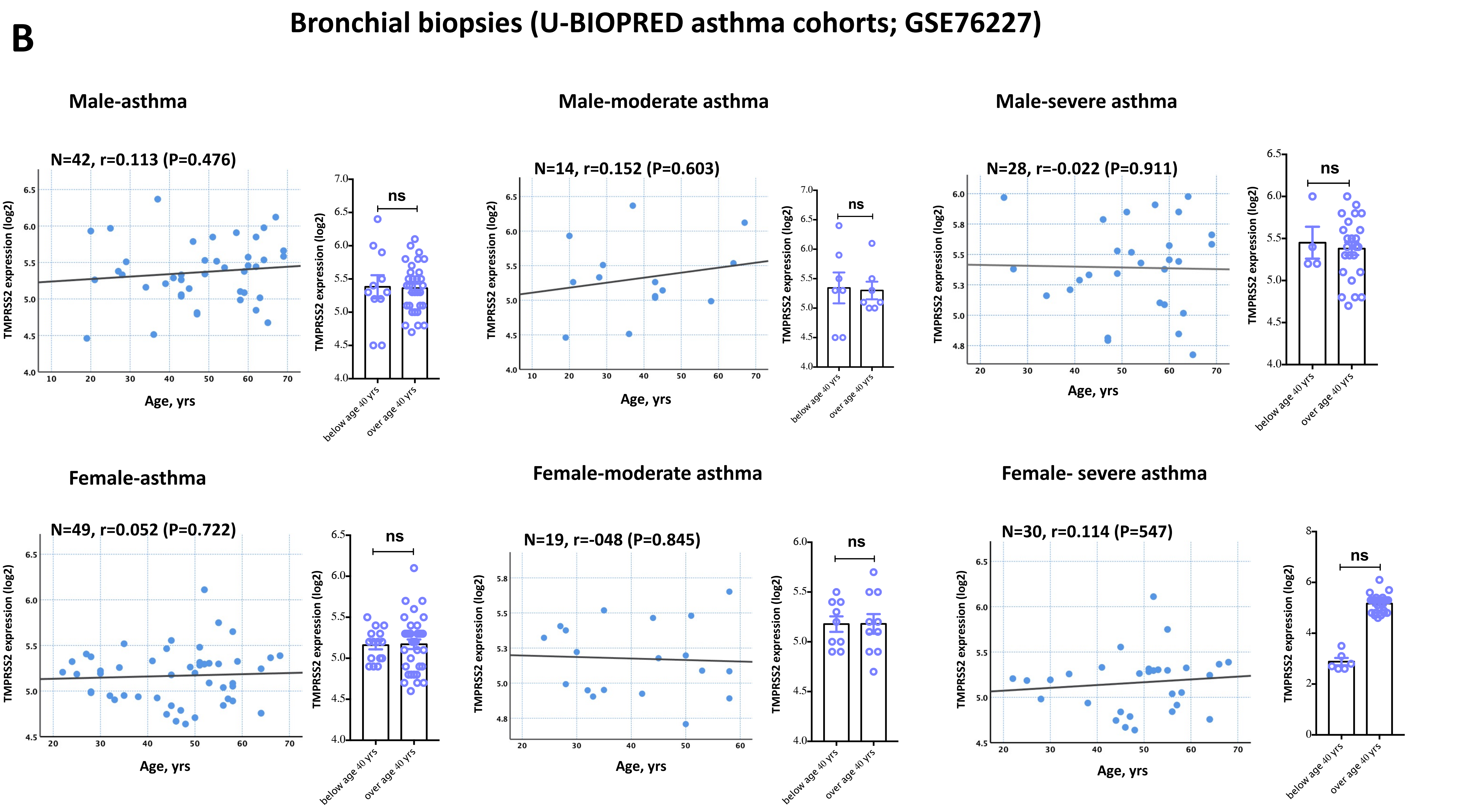
B
Bronchial biopsies (U-BIOPRED asthma cohorts; GSE76227)
Male-asthma
N=42, r=0.113 (P=0.476)
TMPRSS2 expression (log2)
Age, yrs
ns
below age 40 yrs
over age 40 yrs
Male-moderate asthma
N=14, r=0.152 (P=0.603)
TMPRSS2 expression (log2)
Age, yrs
ns
below age 40 yrs
over age 40 yrs
Male-severe asthma
N=28, r=-0.022 (P=0.911)
TMPRSS2 expression (log2)
Age, yrs
ns
below age 40 yrs
over age 40 yrs
Female-asthma
N=49, r=0.052 (P=0.722)
TMPRSS2 expression (log2)
Age, yrs
ns
below age 40 yrs
over age 40 yrs
Female-moderate asthma
N=19, r=-048 (P=0.845)
TMPRSS2 expression (log2)
Age, yrs
ns
below age 40 yrs
over age 40 yrs
Female- severe asthma
N=30, r=0.114 (P=547)
TMPRSS2 expression (log2)
Age, yrs
ns
below age 40 yrs
over age 40 yrs

Supplementary Figure 1. Gene expression of TMPRSS2 in airway epithelium (A) and bronchial biopsies (B) of moderate and severe asthmatics relative to gender. Correlation between ACE2 gene expression level and individual's age was measured using Pearson's correlation coefficient with a two-sided test for significance ($P<0.05$ significant). Two-way comparison was done using unpaired t-test or Mann-Whitney U test, depending on the skewness of the data. ns: non-significant. * $P<0.05$

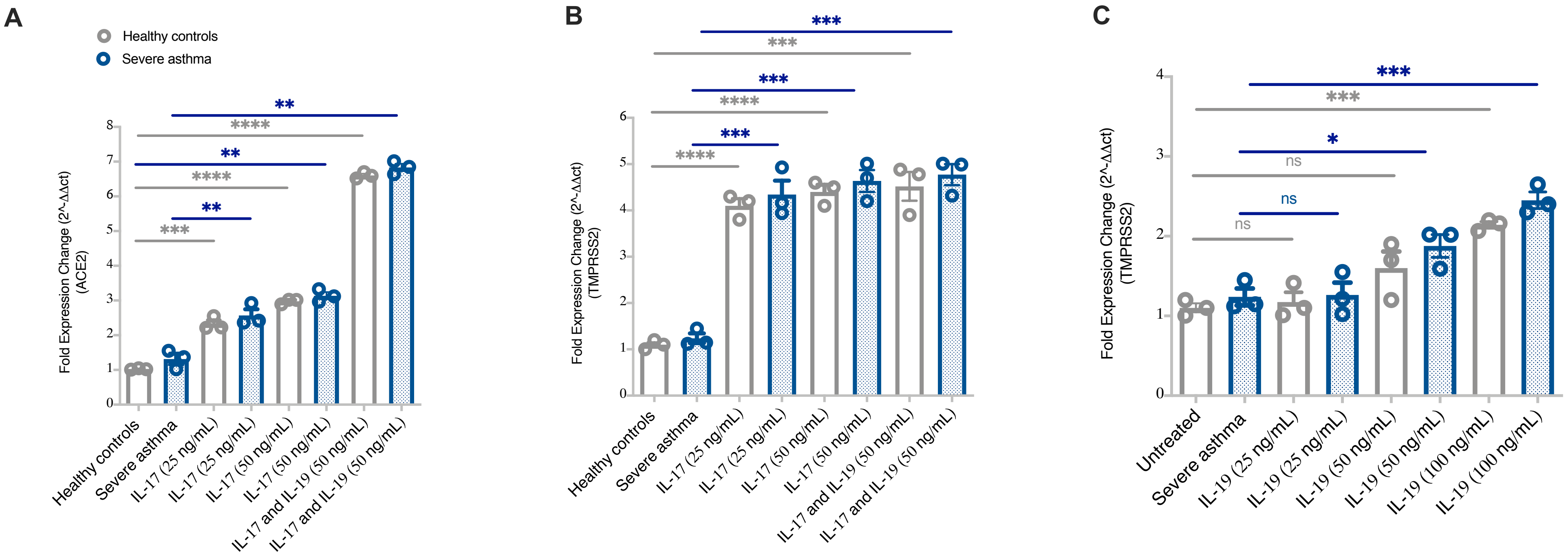

Supplementary Figure 2. ACE2 (A) and TMPRSS2 (B and C) mRNA expression in primary human bronchial fibroblasts (n=3) following in vitro stimulation with different doses of IL-17 and/or IL-19 cytokines. Two-way comparison was done using unpaired t-test or Mann-Whitney U test, depending on the skewness of the data. *** P<0.001.

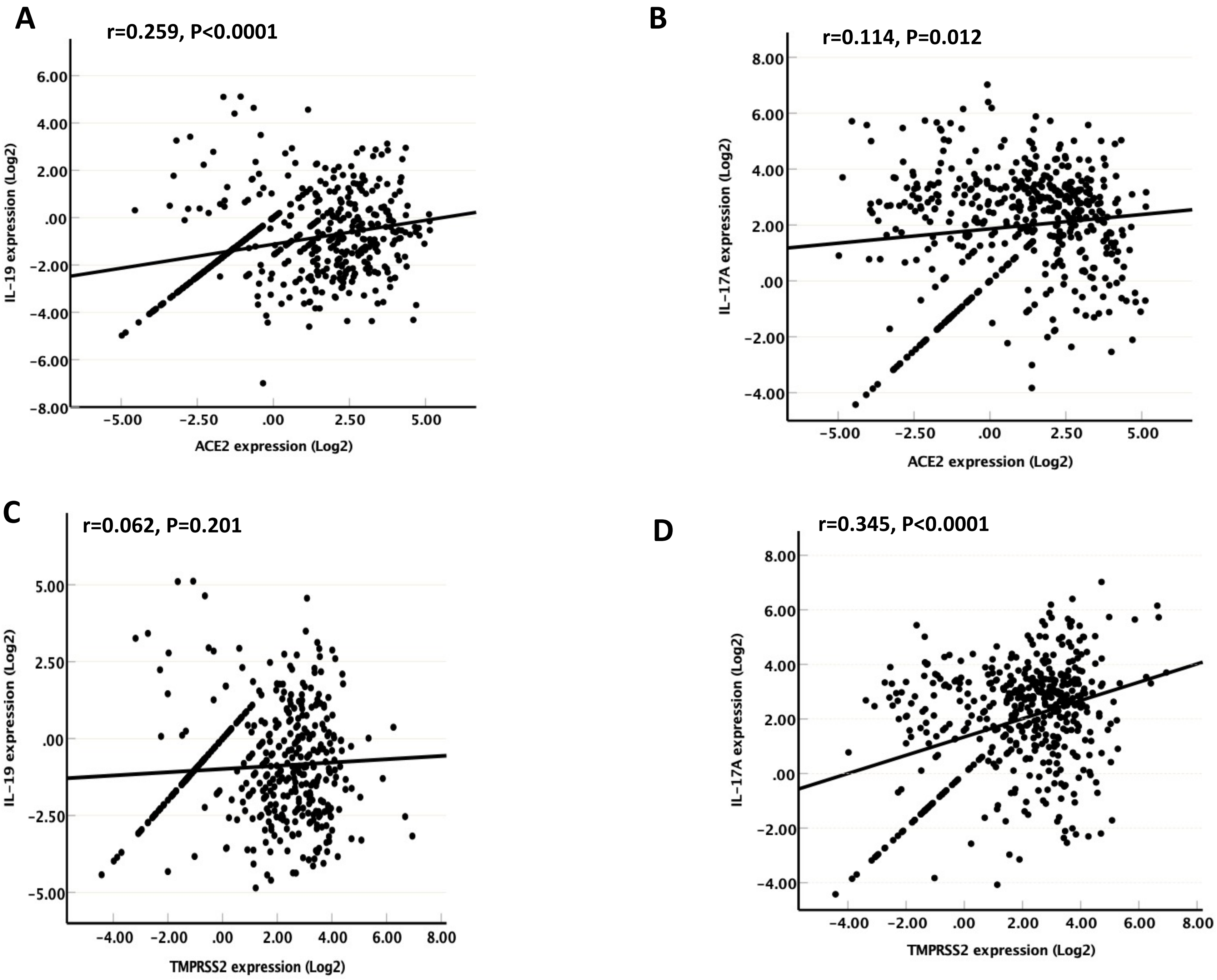

A
r=0.259, P<0.0001
IL-19 expression (Log2)
ACE2 expression (Log2)
B
r=0.114, P=0.012
IL-17A expression (Log2)
ACE2 expression (Log2)
C
r=0.062, P=0.201
IL-19 expression (Log2)
TMPRSS2 expression (Log2)
D
r=0.345, P<0.0001
IL-17A expression (Log2)
TMPRSS2 expression (Log2)

Supplementary Figure 3. Correlation between IL-19 or IL-17 and ACE2 or TMPRSS2 gene expression levels in nasopharyngeal swabs of COVID-19 patients (n=430 COVID-19 patients; GSE152075). Pearson's correlation coefficient with $P<0.05$ considered significant; two-sided test.

Airway epithelium (SARP asthma cohorts; GSE43696)

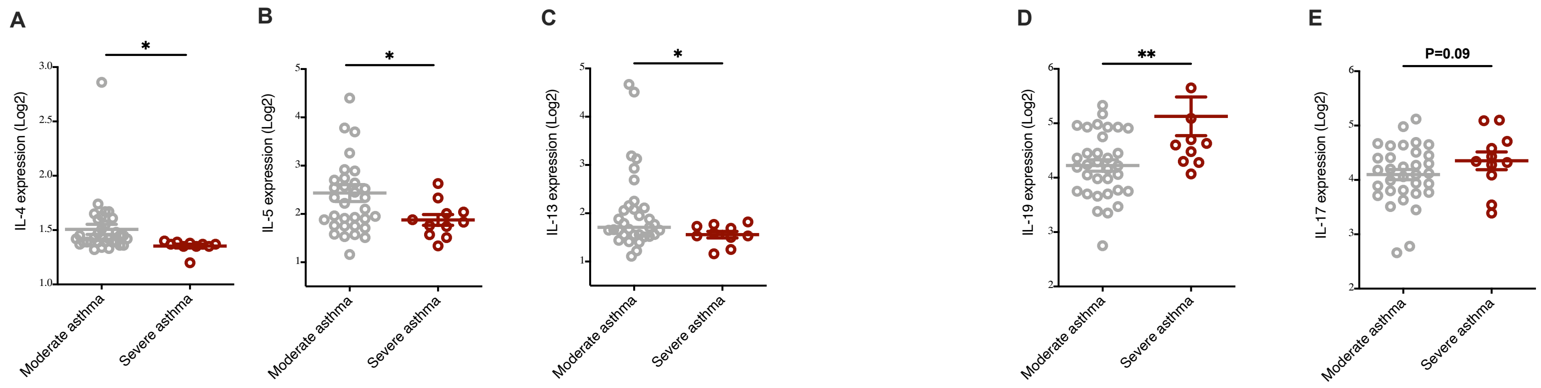

Bronchial biopsies (U-BIOPRED asthma cohorts; GSE76227)

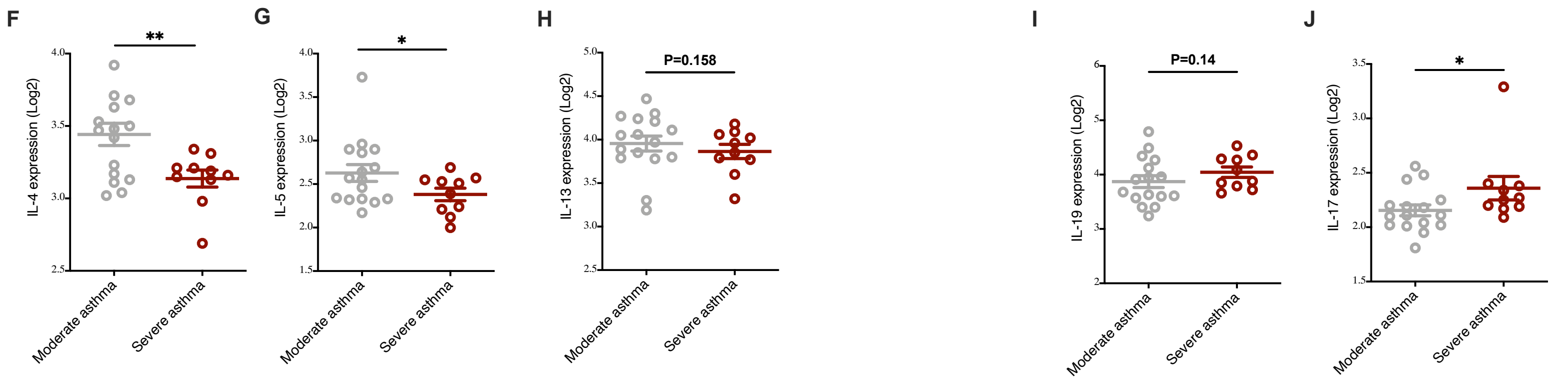

Supplementary Figure 4. The gene expression levels of type II cytokines (IL-4, IL-5, and IL-13), IL-19, and IL-17 in airway epithelium and lung tissues of asthmatics younger than 40 years of age. Two-way comparison was done using unpaired t-test or Mann-Whitney U test, depending on the skewness of the data. *** P<0.001.
